# Catalytic Reduction of H_2_O_2_ by Polyvinylpyrrolidone Nickel Oxide Nanozymatic Activity and Colorimetric Sensing of Ascorbic Acid

**DOI:** 10.3390/bios16050299

**Published:** 2026-05-21

**Authors:** Mosebudi Rambevha, Ridge Chavalala, Philani Mashazi

**Affiliations:** Institute for Nanotechnology Innovation, Rhodes University, P.O. Box 94, Makhanda 6140, South Africa; g24r5071@campus.ru.ac.za (M.R.); g17c9530@campus.ru.ac.za (R.C.)

**Keywords:** polyvinylpyrrolidone, nickel oxide nanoparticles, nanozymes, colorimetric sensor, ascorbic acid

## Abstract

Ascorbic acid (AA) or vitamin C is an important biomolecule that plays a crucial role in biological and physiological systems. Deficiency and/or excess of AA in the body can lead to severe diseases such as scurvy and gastrointestinal complications. Therefore, it is crucial to monitor the levels of AA in the body and supplements. Polyvinylpyrrolidone nickel oxide nanoparticles (PVP-NiONPs) are prepared and evaluated for their potential as nanozymes with peroxidase-like activity. o-Phenylenediamine (OPD) was used as a chromogen in the presence of hydrogen peroxide. The oxidized OPD was produced by ROS from PVP-NiONPs and H_2_O_2_. This was monitored using UV-vis spectra and by colour changes using the naked eye. AA reduced the oxidized OPD during its sensing. The UV-vis signal was linear for AA concentrations ranging from 40 µM to 400 μM. The limit of detection (LOD) for AA was calculated to be 0.11 μM using 3σ and the limit of quantification (LOQ) was 0.36 μM using 10σ indicating a very high sensitivity. The colorimetric sensor showed good reproducibility and a recovery rate between 92.3% and 102.6%, indicating high accuracy and reliability. The findings of this work confirmed that PVP-NiONPs possess enzyme-like activity and are a promising alternative for the quantitative, on-site detection of ascorbic acid.

## 1. Introduction

Ascorbic acid (AA), commonly known as vitamin C, is an essential water-soluble biomolecule. It plays a crucial role in biological and physiological processes. It acts as an antioxidant and an immune booster. AA plays a role in the synthesis of collagen and regulation of neurotransmitters [1,2,3]. The antioxidant properties of AA allow it to scavenge reactive oxygen species (ROS). Therefore, it is able to relieve biological cells from oxidative stress and the associated damages. Deficiency and/or excess levels of AA can result in serious health complications. vitamin C deficiency is related to anemia, scurvy and slow wound healing. Excess Vitamin C intake is associated with gastrointestinal complications and elevated risks of kidney stones. Therefore, it is critical to maintain and accurately monitor the levels of AA in clinical, pharmaceutical and food products [4].

There are many methods employed for the detection of ascorbic acid and these include high-performance liquid chromatography (HPLC) [5,6], capillary electrophoresis [7], redox titration [8,9,10] and electrochemical methods [11,12,13,14,15]. These conventional methods offer high accuracy, selectivity and sensitivity but require complex instrumentation, time-consuming sample preparations and highly skilled operators. These limitations have necessitated the need for alternative methods that are simple to use, low-cost and rapid. As a result, colorimetric sensors which allow for visual detection through colour changes and measurable signals have emerged as promising tools [16,17,18,19].

Nanostructured metal oxides have received research attention due to myriad applications. They have been shown to possess peroxidase-like activity [20,21,22]. Nickel oxide nanoparticles (NiONPs) have been widely evaluated as catalysts with a tuneable band gap, large surface area and strong electron transfer abilities [23,24].

In this work, polyvinylpyrrolidone (PVP)-stabilized NiONPs are prepared and evaluated for their properties as a catalytic reactive oxygen radical species (ROS) generator. The ROS generation ability will be utilized for the oxidation of OPD resulting in a colour change. Ascorbic acid, when introduced into this catalytic system, acts as a mild reducing agent. It reduces the oxidized OPD resulting in its colorimetric detection. In this way, ascorbic acid is quantified [25,26]. The use of PVP-NiONPs for AA detection is reported here for the first time.

## 2. Experimental

### 2.1. Material and Reagents

Potassium hydroxide (KOH), sodium hydroxide (NaOH), nickel (II) nitrate hexahydrate (Ni(NO_3_)_2_·6H_2_O), ethanol (EtOH), hydrogen peroxide (H_2_O_2_), sodium acetate (NaOAc), o-phenylenediamine (OPD), acetic acid (CH_3_COOH), polyvinylpyrrolidone (PVP), isopropanol (IPA), dimethyl sulfoxide (DMSO), terephthalic acid (TA), ethylenediaminetetraacetic acid (EDTA), p-benzoquinone (PBQ), ascorbic acid (AA), 32% hydrochloric acid (HCl), sucrose (Suc), fructose (Fru), lactose (Lac), fucose (Fuc), galactose (Gala), glucose (Glu), cysteine (Cys), glycine (Gly), lysine (Lys), glutathione (Glut), urea, uric acid (UA), glycolic acid (Gla), magnesium sulphate (MgSO_4_), potassium chloride (KCl), sodium chloride (NaCl), adenine (Ade, A), guanine (Gua, G), thymine (Thy, T) and cytosine (Cyt, C) were all purchased from Sigma-Aldrich South Africa. All the reagents were of analytical grade. Vitamin C tablets were purchased from Alpha Pharm Grahamstown. All aqueous solutions were prepared using ultrapure water with a resistivity of 18.2 ΩM.cm obtained using Elga LabWater laboratory water purification (Elga Veolia) was purchased from ELGA LabWater, UK. Acetate buffer (0.20 M) and phosphate-buffered saline (0.15 M) solutions were prepared following AAT Bioquest. The aqueous solutions were mixed into a 1 L volumetric flask. The pH of the solutions was adjusted by NaOH (1.0 M) or HCl (1.0 M) to the desired pH values. OPD is a carcinogenic compound. Care must be taken when working with it; that is, work should be undertaken in a well-ventilated laboratory with appropriate PPE worn at all times and proper chemical waste disposal must be strictly followed.

### 2.2. Apparatus

UV-vis spectroscopy measurements were carried out using a ThermoFischer Scientific South Africa, Multiskan Sky with Cuvette and Touch screen, 100–240 V. Fourier Transform Infrared (FTIR) spectroscopy measurements were carried out using a PerkinElmer spectrum 100 FTIR spectrometer purchased from PerkinElmer South Africa, Midrand. The infrared spectra were recorded between 500 and 4000 cm^−1^. The measurement of nanoparticle size was carried out using a Malvern Zetasizer Nano-ZS90 series equipped with a 633 nm He/Ne laser for dynamic light scattering (DLS) purchased from Malvern Panalytical UK. X-ray diffraction (XRD) patterns were obtained with a BRUKER D8 Advance X-ray diffractometer purchased from Bruker AXS GmbH, Karlsruhe Germany using the *Cu Kα* radiations (*λ* = 1.54059 Å). Data was collected in the angular region of 2*θ* = 20–90° at room temperature in a step-scanning mode, with a step length of 0.02° to obtain the crystallinity of nanoparticles. The particle sizes were calculated using the Debye–Scherrer Equation (1) and the XRD peak at (200):

(1) $$D=\frac{K\lambda \;}{\beta Cos\theta }$$

where *D* is the nanoparticle crystalline size, *K* represents the Scherrer constant (0.98), λ denotes the wavelength (1.54), and *β* denotes the full width at half maximum (FWHM).

A BRUKER Vertex 70-Ram II Raman spectrometer purchased from Bruker, Germany (equipped with a 1064 nm Nd:YAG laser and liquid nitrogen-cooled germanium detector) was used to collect Raman spectral data. Transmission Electron Microscopy (TEM) micrographs were obtained using a Zeiss Libra 120 TEM operating at 80 kV purchased from Zeiss, Oberkochen Germany. PVP-NiONPs were dissolved in water, dropped onto a carbon-coated copper grid and allowed to dry overnight at room temperature before measurement.

### 2.3. Preparation of PVP-NiONPs

The preparation of PVP-NiONPs was achieved using a hydrothermal method. Briefly, nickel (II) nitrate hexahydrate (2.0 g, 6.8 mmol) and 1.0 g of PVP were added into 50 mL ultrapure water and stirred at room temperature for 10 min or until a homogenous solution was achieved. A 50 mL aqueous solution of NaOH (0.20 g, 5.0 mmol) was added dropwise and the solution was murky. The slurry mixture was then transferred into a high-pressure autoclave reactor. The autoclave was sealed, and the mixture was hydrothermally treated at 120 °C for 12 h with stirring. The autoclave was then allowed to cool to room temperature. The precipitate was then collected by centrifugation and washed several times with ethanol and deionized water. After filtration, the precipitate (PVP-NiONPs) was left to dry overnight in an oven. The dried green precursor was transferred to a ceramic crucible and calcined in a furnace at 400 °C for 3 h. The black powder that resulted was collected to yield PVP-NiONPs.

### 2.4. ROS Generated by PVP-NiONPs and H_2_O_2_ Oxidize OPD

The oxidation of OPD by ROS (generated by PVP-NiONPs and H_2_O_2_) was investigated at room temperature (25 °C). Briefly, PVP-NiONPs (2.0 mg/mL, 400 μL), H_2_O_2_ (0.10 mM, 200 μL), and OPD (3.20 mM, 100 μL) in acetate buffer (pH 4.0, 0.20 M, 2.30 mL) were incubated together for 10 min. An orange- or yellow-coloured solution resulted and was monitored using a UV-vis spectrophotometer and the absorption peak of oxidized OPD was observed at 450 nm.

### 2.5. Optimizing of Conditions of Enzyme-like Activity of PVP-NiONPs

#### 2.5.1. Effect of pH on Oxidation of OPD

The absorption of oxidized OPD at 450 nm was monitored in the presence of PVP-NiONPs and H_2_O_2_ in acetate buffer with different pH values. Briefly, OPD (0.10 mM), PVP-NiONPs (0.27 mg/mL) and H_2_O_2_ (0.10 mM) were added together in acetate of different pH values from pH 2.0 to pH 8.0. In each of the pH solutions, the concentrations of PVP-NiONPs, OPD and H_2_O_2_ were the same. The reaction time of 5 min and temperature (25 °C) were kept constant.

#### 2.5.2. Effect of Time on Oxidation of OPD

The oxidation of OPD was investigated over a period of time. For this investigation, the OPD (0.10 mM), PVP-NiONPs (0.27 mg/mL) and H_2_O_2_ (0.10 mM) in pH 4.0 (0.20 M) acetate buffer was allowed to react over time up to 30 min. The oxidation of OPD was measured. The reaction concentrations of PVP-NiONPs, OPD, and H_2_O_2_ were the same. The temperature (25 °C) was also kept constant.

#### 2.5.3. Effect of Temperature on Oxidation of OPD

For PVP-NiONPs, the effect of temperatures was investigated by monitoring the oxidized OPD absorbance at different temperatures. The concentration of OPD (0.10 mM), PVP-NiONPs (0.27 mg/mL) and H_2_O_2_ (0.10 mM) in pH 4.0 (0.20 M) acetate buffer were kept the same and used to study the change in absorbance at 450 nm in different temperatures ranging from 20 °C to 70 °C. The reaction time of 5 min was kept constant.

#### 2.5.4. Effect of the H_2_O_2_ Concentration on Oxidation of OPD

The initial rates (*V*_0_) of decreasing absorption of OPD were monitored against the changes in concentration of H_2_O_2._ The concentration of PVP-NiONPs (0.27 mg/mL), OPD (0.10 mM) and acetate buffer (pH 4.0, 0.20 M) were kept the same. The concentrations of H_2_O_2_ studied ranged from 2.0 to 20 mM. The reaction time of 5 min and temperature (25 °C) were kept constant.

#### 2.5.5. Effect of the OPD Concentration

The changes in concentration and initial rates (*V*_0_) of OPD absorption were measured. The concentration of PVP-NiONPs (0.27 mg/mL), H_2_O_2_ (0.10 mM) and pH 4 (0.20 M) acetate buffer were kept the same. The OPD concentrations investigated ranged from 0.20 to 2.0 mM. The reaction conditions such as the reaction time of 5 min and temperature (25 °C) were also kept constant.

#### 2.5.6. ROS Confirmation Using Radical Scavengers

The enzyme-mimetic properties of PVP-NiONPs were evaluated using reactive oxygen radical species scavengers. To identify the contribution of reactive oxygen radical species, generated EDTA, IPA and PBQ were used. EDTA captures holes (h^+^) due to photoexcitation, IPA is selective toward hydroxyl radicals (HO^●─^) and PBQ is a superoxide radical (O_2_^●─^) scavenger. The investigation relies on the oxidation of OPD to produce a yellow-coloured chromophore with absorbance at 450 nm. The effect of different scavengers on the reaction was monitored using UV-vis spectroscopy. Experimentally, in four 1.5 mL vials using acetate buffer (pH 4.0) as medium, PVP-NiONPs (2.0 mg/mL, 400 μL) and OPD (200 µL of 1.0 mM) were added. This was followed by the addition of 300 μL solution of H_2_O_2_ (2.0 mM) and 100 µL of radical scavengers (EDTA or IPA or PBQ). The control experiment was without radical scavengers, whilst the three other vials contained IPA to scavenge hydroxyl radicals (HO^●─^), PBQ as a superoxide radical (O_2_^●─^) scavenger, and the last 1.5 mL vial contained EDTA to scavenge electron holes (h^+^). The changes in absorbance at 450 nm were monitored using a UV-vis spectrophotometer over a fixed time interval. For each of the scavengers, EDTA or IPA or PBQ, the experiments were done in triplicates to ensure reproducibility.

#### 2.5.7. Detection of Ascorbic Acid, Scheme 1

**Scheme 1:**
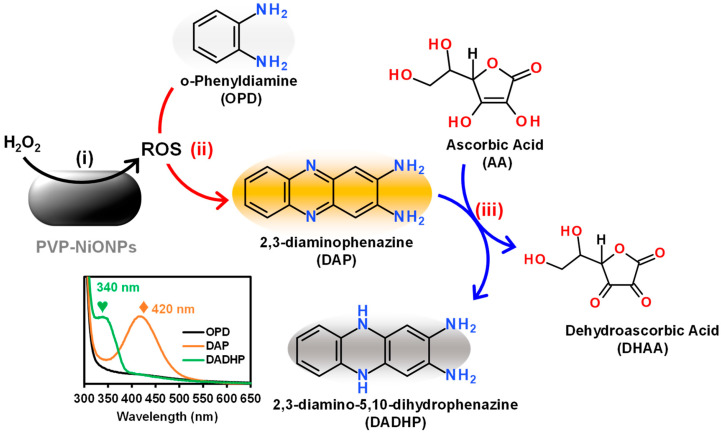
(i) Production of ROS by PVP-NiONPs, (ii) oxidation of DAP by ROS, (iii) oxidation of AA to form DHAA and reduction of DAP to form DADHP.

Ascorbic acid is a mild reducing agent, and this property has been used in the preparation of metal nanoparticles [27]. Scheme 1 shows the mechanistic illustration of the detection of ascorbic acid through the OPD/H_2_O_2_ catalytic reaction in the presence of PVP-NiONPs. Here, PVP-NiONPs catalytically reduce H_2_O_2_ to form reactive oxygen radical species resulting in the oxidation of OPD to form DAP. The presence of AA in the same solution reduces DAP to form 2,3-diamino-5,10-dihydrophenazine (DADHP). The increasing optical signal was monitored as a function of varied concentration of AA for colorimetric sensing. Ascorbic acid standards with concentrations from 40 μM to 400 μM were prepared and analyzed. To conduct this investigation, PVP-NiONPs were dispersed in acetate buffer (pH 4.0) followed by the addition of a substrate (H_2_O_2_ and OPD). Different concentrations of AA were immediately added into the solution. H_2_O_2_ acted as the fuel of the reaction. The reaction was allowed to react for 5 min. Colour change was observed and monitored using a UV-vis spectrophotometer.

#### 2.5.8. Selectivity, Reproducibility, and Applications

The selectivity of the proposed PVP-NiONP-based sensor for ascorbic acid was investigated against closely related species. The compounds were added in the solution following the method in Section 2.5.7 but AA was substituted by the interfering species. The investigated interfering compounds included common sugars such as sucrose (Suc), fructose (Fru), lactose (Lac), fucose (Fuc), galactose (Gala) and glucose (Glu), and amino acids such as cysteine (Cys), glycine (Gly), lysine (Lys) and glutathione (Glut). Organic compounds and inorganic salts such as urea, uric acid (UA), glycolic acid (GLA), magnesium sulphate (MgSO_4_), potassium chloride (KCl), and sodium chloride (NaCl) were evaluated. Nucleobases were also investigated and these are adenine (Ade, A), guanine (Gua, G), thymine (Thy, T), and cytosine (Cyt, C). The reproducibility was evaluated by the measuring of three consecutive detections. The measurements were performed on the same day and during the same laboratory experiment under the same conditions. Percentage relative standard deviation (%RSD) values were calculated. Furthermore, the sensor was applied for ascorbic acid detection in vitamin C supplements as a representative of real samples. The 10% vitamin C was spiked with known concentrations of ascorbic acid (120 µM, 160 µM, 200 µM). The optical signal change was investigated and quantified using the ascorbic acid calibration curve.

## 3. Results and Discussion

### 3.1. Characterization of PVP-NiONPs

**Figure 1:**
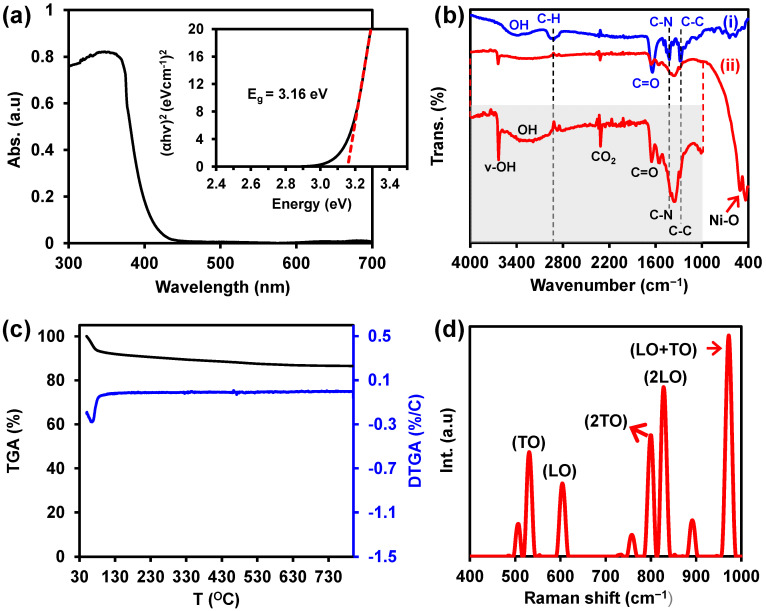
(**a**) UV-vis spectrum with fitted Tauc plot (inset), (**b**) FTIR spectra of (i) PVP and (ii) PVP-NiONPs, (**c**) thermogram with its derivative, and (**d**) Raman spectrum of PVP-NiONPs.

UV-vis absorption spectroscopy was used to investigate the optical characteristics of the PVP-NiONPs. The spectrum in Figure 1a shows an absorption peak at around 347 nm. The optical band gap energy of the prepared PVP-NiONPs was accessed using the Tauc Equation (2) [28].

(2) $${\left(\alpha hv\right)}^{2}=B\left(h\nu -E_{g}\right)$$

where α is the absorption coefficient, *h*ν is the photon energy, *E*_g_ is the band gap energy, and *B* is a constant.

From Equation (2), a plot of (*αhv*)^2^ vs. photon energy (*hv*, eV) was obtained. A linear portion of the Tauc plot was extrapolated to the photon energy axis to estimate the band gap which was found to be 3.16 eV. This suggests the electrons in the PVP-NiONPs move directly from the valence band to the conduction band without any change in momentum. This transition makes the PVP-NiONPs exhibit strong ultraviolet (UV) light absorption that enhances their optical and catalytic ideal for colorimetric sensing. The obtained band gap is in good concordance with previously reported values for NiO nanoparticles which usually range from 3.10 eV to 4.00 eV, depending on the synthetic route, size of the nanoparticles, and their crystallinity. Ahmed et al. [29] reported a band gap of 3.07 eV using computational methods while Patel et al. [30] obtained a bang gap of 3.80 eV for NiO nanofilms prepared using DC reactive sputtering.

The FTIR spectrum in Figure 1b for the PVP-NiONPs was recorded from 4000 cm^−1^ to 400 cm^−1^ to identify functional groups present and to confirm the interaction between PVP as a stabilizing agent and the surface of the NiONPs. A sharp absorption band observed at 3640 cm^−1^ corresponds to the free-base hydroxyl stretching from the nanoparticle surface-adsorbed water molecules [31]. Two overlapping bands at 1650 cm^−1^ are attributed to the C=O vibration band from the pyrrolidone ring. Also, the coexisting broad peaks observed at 1360 cm^−1^ correspond to C-N and C-C bending vibration peaks from the PVP. The presence of typical intense peaks at 500 and 450 cm^−1^ corresponds to stretching vibrations of the cubic NiO lattice [32,33].

Thermogravimetric analysis (TGA) was studied to assess thermal stability of the prepared PVP-NiONPs under nitrogen gas in the range between 50 °C and 800 °C. The PVP-NiONPs decomposed in one step during heat treatment as shown in Figure 1c. The weight loss was approximately 6% between 50 °C and 90 °C attributable to the evaporation of surface moisture as observed in FTIR spectra. At temperatures above 100 °C, the PVP-NiONPs became stable. The residual weight is around 94% at 800 °C confirming high purity and stability of the PVP-NiONPs. The Ni-O ionic bond is very strong and resulted in strong resistance to decomposition even at elevated temperatures. These results show that NiONPs can be used in high temperature applications. Similar observations were found by Salavati et al. [34] where the observed total weight loss was around 40% while Silviya and Mahalakshmi [35] only observed a total weight loss of only 6% with very stable NiONPs.

The Raman spectrum of the as-prepared PVP-NiONPs have peaks characteristic of a cubic crystal structure as shown in Figure 1d. A sharp band at 550 cm^−1^ corresponds to one phonon transverse optical (TO). A band at 600 cm^−1^ corresponds to a longitudinal optical (LO) phonon of the PVP-NiONPs. In bulk NiO, there is no first-order symmetric Raman scattering because of the rock salt. The appearance of first order peaks is due to the nano-size PVP-NiONPs and defects because of phonon confinement lattice strain and also the oxygen vacancies. A peak at 800 cm^−1^ corresponds to second-order two-phonon scattering (2TO mode) which forms due to distortions. The last two peaks of 850^−1^ and 1000 cm^−1^ can be attributed to combination bands or overtones. The combination at 850 cm^−1^ is two longitudinal optical phonons while at 1000 cm^−1^ the combination consists of one longitudinal and one transverse optical phonon similar to the previous report [36]. Defects play a significant role by creating more reactive sites on the surface of the NiONPs [37]. Overall, the Raman spectrum confirms the successful synthesis of PVP-stabilized NiONPs with a wurtzite structure with high crystalline peaks.

**Figure 2:**
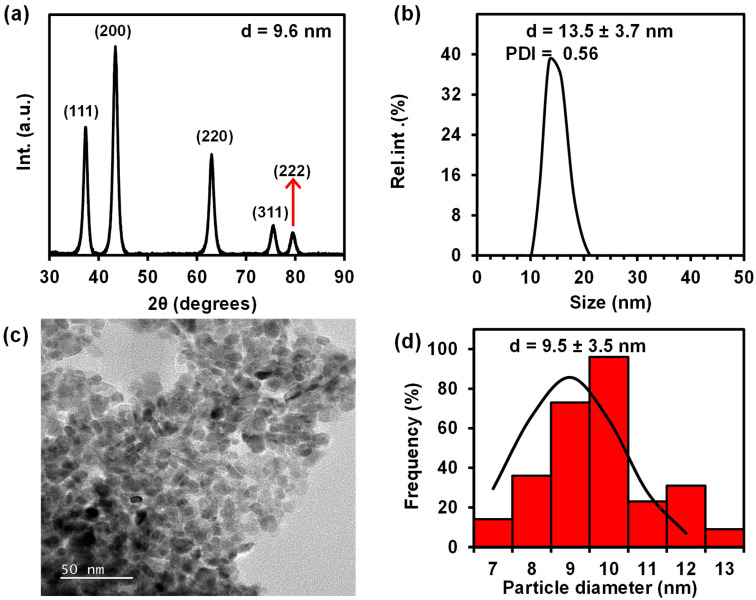
(**a**) XRD pattern, (**b**) particle size from DLS, (**c**) TEM micrographs 50 nm magnification, and corresponding (**d**) particle size distribution histogram obtained from 50 nm magnification.

XRD was used to confirm the crystallinity of PVP-NiONPs in Figure 2a. The Braggs reflections show the crystalline nature of the PVP-NiONPs, and the broad peaks confirm that the prepared material consists of nano-sized particles. The diffraction patterns at the 2θ (degree) values 37.3°, 43.4°, 63.0°, 75.5°, and 79.5° correspond to (111), (200), (220), (311) and (222) Miller Indices, respectively. The diffraction pattern and their corresponding Miller Indices of the as-prepared PVP-NiONPs were similar to those reported in the literature [38,39,40] and match the Joint Committee on Powder Diffraction Standards (JCPDS Card No. 47–1049) with the lattice constants *a* = 3.249 Å and *c* = 5.206 Å of the pure face-centred cubic (FCC) structure of PVP-NiO [41]. There are no additional peaks in the XRD pattern confirming that the NiONPs are pure. The average particle size of 9.6 nm of the PVP-NiONPs was determined from the broadening of the peak at the 43.4° most intense peak (200) in the XRD pattern using Debye–Scherrer’s equation.

The DLS in Figure 2b shows PVP-NiONPs in solution with narrow particle sizes found to be 13.5 ± 3.7 nm. The obtained Polydispersity Index (PDI) on DLS is a measure of the size distribution of PVP-NiONPs in a suspension and was found to be 0.56. The range between 0.1 and 0.3 means that the particles are monodisperse. The PDI was more than 0.3 confirming that the particles were moderately polydisperse [42]. There was a slight difference between the calculated particle sizes from XRD and DLS, in that DLS measures hydrodynamic diameter which includes the solvating layer. There is a clear indication of large and small particles co-existing in the suspension and DLS is more sensitive to larger particles as shown by PDI. According to the study of Rehman et al. [43] it was found that using a stabilizing/capping agent can actually increase the PDI value which was the case for PVP-NiONPs with PVP as a stabilizing agent.

The morphology and size of the nanoparticles was further evaluated using TEM as shown with different magnifications of 50 nm and 10 nm. The TEM image with magnification at 50 nm in Figure 2c is densely packed with clustered ultra-small PVP-NiONPs. The PVP-NiONPs consist of a mixture of oval and some spherical particles. A histogram in Figure 2d shows the nanoparticle sizes were found to be 9.5 ± 3.5 nm. The TEM nanoparticle size was in concordance with the particle size estimated from XRD using the Scherrer equation.

### 3.2. Peroxidase-like Activity of PVP-NiONPs

**Figure 3:**
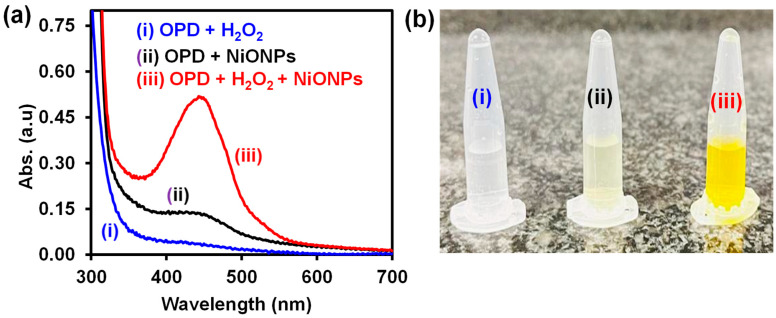
(**a**) UV-vis spectra of OPD in the presence of (i) H_2_O_2_, (ii) PVP-NiONPs, and (iii) H_2_O_2_ and PVP-NiONPs, and (**b**) corresponding images of the reactions.

The peroxidase-like activity of PVP-NiONPs was investigated by the oxidation of OPD in the presence of H_2_O_2_. The UV-vis spectrum in Figure 3a shows absorption spectra of (i) H_2_O_2_ and OPD, (ii) OPD and PVP-NiONPs, and (iii) OPD, H_2_O_2_ and PVP-NiONPs in pH 4.0 acetate buffer. In a solution containing OPD with H_2_O_2_ alone the solution remained clear, and no absorption was observed. The UV-vis spectrum of OPD containing PVP-NiONPs and H_2_O_2_ showed the absorption peak at 450 nm. This is due to the oxidation of OPD to form 2,3-diaminophenazine (DAP). A similar observation was discovered in the reaction system oxidoperoxido molybdenum (VI) complex with OPD and H_2_O_2_ [44]. The colour of the solution turned dark yellow at pH 4.0 buffer solution as seen in Figure 3b and this confirms that PVP-NiONPs possess peroxidase-like activity. Both OPD and H_2_O_2_ are necessary for the peroxidase-like activity of PVP-NiONPs. This behaviour was similar to the commonly used horse-radish peroxidase (HRP) enzyme with OPD and H_2_O_2_ in the similar solution. The biomolecule HRP requires reaction conditions to be controlled. NiONPs possess enzyme-like activity that can be optimized for enhanced signal by changing the experimental conditions.

### 3.3. Conditions Affecting Peroxidase-like Activity of PVP-NiONPs

**Figure 4:**
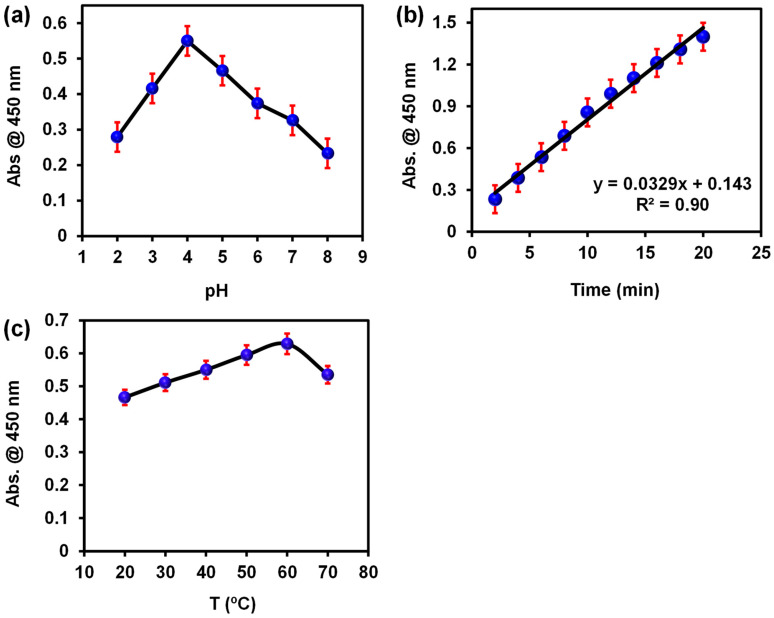
Effect of (**a**) pH, (**b**) reaction time, and (**c**) temperature on the peroxidase-like activity of PVP-NiONPs in a solution containing OPD and H_2_O_2_.

Similar to natural enzymes, the peroxidase-like activity of PVP-NiONPs as artificial enzymes can be affected by different reaction conditions. The effect of pH, reaction time and temperature were evaluated in a reaction system containing OPD, PVP-NiONPs and H_2_O_2_. The effect of pH conditions was assessed in acetate buffers with pH values ranging from pH 2.0 to pH 8.0, as seem in Figure 4a. The OPD absorption in a solution containing PVP-NiONPs and H_2_O_2_ was recorded and the peak at 450 nm was observed and due to OPD oxidation as seen in Figure S1a. The optimum pH value of 4.0 had highest absorption intensity and the most intense yellow colour. The solution colour fading from yellow to colourless was observed at pH 7.0 and pH 8.0. In the basic solution, hydrogen peroxide decomposes to form water and oxygen. Vallabi et al. [45] and Drozd et al. [46] showed similar results for peroxidase-like activity of iron oxide and gold nanoparticles, respectively. In both cases, the acidic pH conditions exhibited the highest relative activity.

The effect of the reaction time ranging from 2 to 20 min and the absorbance at 450 nm for the OPD oxidation (PAD) was investigated. The UV-vis spectra in Figure S1b shows the increasing absorption at 450 nm as the reaction was allowed to react for a long reaction time. The graph of the absorbance intensity at 450 nm increased linearly with time as shown in Figure 4b. Allowing for the reaction to take longer also resulted in the increase in colour intensity of the working solutions from yellow to orange. The regression line in Figure 4b shows excellent linearity, with a correlation coefficient (R^2^) of 0.990, which is very close to 1 suggesting a very close relationship between variables. The reaction time of 5 min of absorption of OPD at 450 nm was chosen for quick analysis.

The effect of the change in temperature, ranging from 20 °C to 70 °C, for the reaction system with OPD, PVP-NiONPs and H_2_O_2_, was investigated. The UV-vis spectra were recorded as shown in Figure S1c. The graph of absorbance at 450 nm versus temperature is shown in Figure 4c and it increased from 20 °C to 60 °C and it decreased afterwards. As the temperature increased, H_2_O_2_ became unstable and converted to water vapour and oxygen.

### 3.4. Effect of Substrate (OPD and H_2_O_2_) Concentration

**Figure 5:**
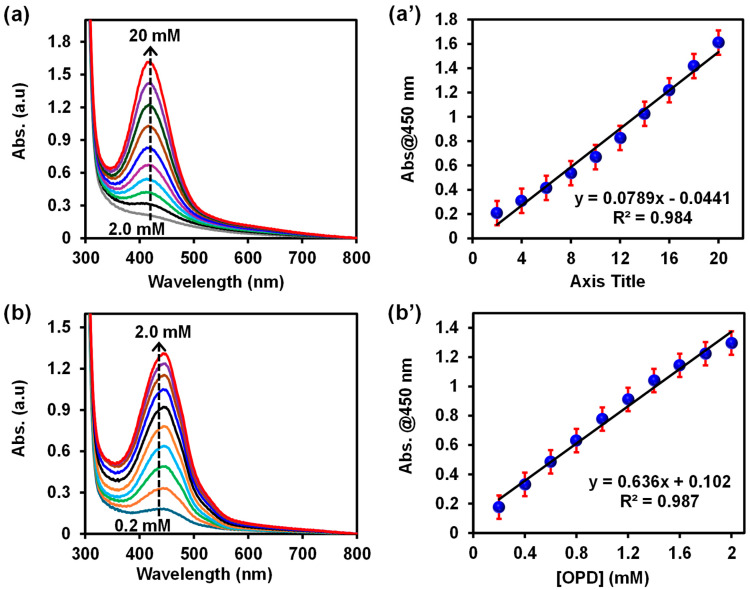
UV-vis spectra of (**a**) H_2_O_2_ and (**b**) OPD showing the effect of changing concentrations and the corresponding calibration curves (**a’**) and (**b’**) of peroxidase-like activity of PVP-NiONPs. Dotted arrows shows increasing concentrations of (**a**) H_2_O_2_ and (**b**) OPD.

The effect of the changes in concentrations of H_2_O_2_ and OPD were evaluated at pH 4.0 for 5 min of reaction time. For H_2_O_2_, the absorbance at 450 nm for the oxidation of OPD was investigated. The concentration of H_2_O_2_ investigated was from 2.0 mM to 20 mM. The absorbance values at 450 nm increased with the increase in concentration of H_2_O_2_ as shown in Figure 5a. The increase in the absorption of OPD at 450 nm was also linear with the increase in H_2_O_2_ concentration, as shown in Figure 5(a’). This observation confirms that the catalytic activity of PVP-NiONPs is significantly affected by the concentration of H_2_O_2_. The H_2_O_2_ concentration of 2.0 mM was chosen for further studies. This phenomenon was also observed in the study of Chen et al. [17] where the catalytic activity was increasing rapidly with increased concentrations of H_2_O_2_ up to 8.0 mM. In this work, the increase in H_2_O_2_ concentration was up to 20 mM which is much higher than the value reported by Chen et al. [17]. The concentration of OPD investigated ranged from 0.20 mM to 2.0 mM as shown in Figure 5b. An increase in the oxidation of OPD as the concentration increased was observed as shown in Figure 5(b’). Similarly, the change in absorption at 450 nm increased with increasing concentration of OPD. This observation confirms that the catalytic activity of PVP-NiONPs is affected by the concentrations of the OPD substrate.

### 3.5. Steady-State Kinetic Analysis of PVP-NiONPs

The steady-state kinetic parameters of PVP-NiONPs were evaluated using the Michaelis–Menten and Lineweaver–Burk (double reciprocal) models. The analysis was performed by varying individual substrate concentration while keeping others constant. H_2_O_2_ and OPD are the components of the substrate solution. The initial rates (*V*_0_) were calculated from the linear region obtained from the time-dependent absorption value at 450 nm. The absorbance values were converted to the concentration using the Beer–Lambert law and the molar absorption coefficient (ԑ = 16,700 M^−1^.cm^−1^) of the oxidized OPD.

**Figure 6:**
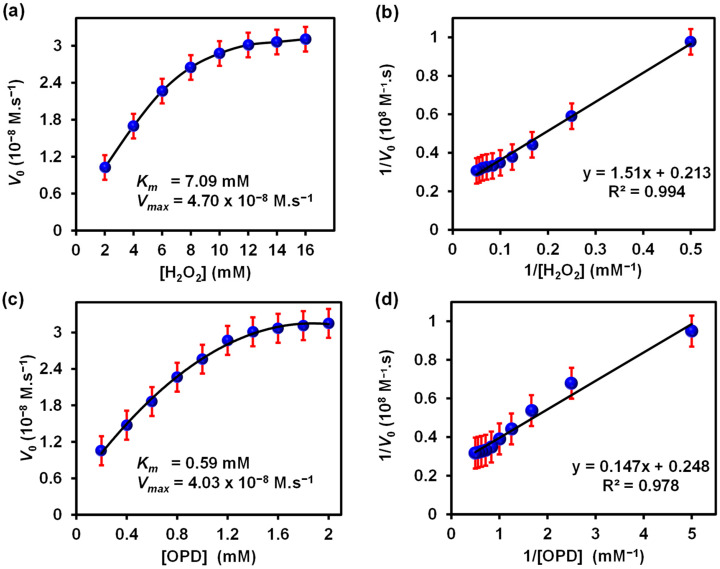
Michaelis–Menten kinetic plots of initial velocity versus the concentration of substrates. (**a**) H_2_O_2_ and (**b**) its double-reciprocal plot, (**c**) OPD and (**d**) its double-reciprocal plot.

Figure 6a showed an increase in *V*_0_ with the increasing concentration of H_2_O_2_. The graph reached a plateau at a high concentration ≥ 12 mM. This confirmed the typical Michaelis–Menten enzyme-like behaviour for the PVP-NiONPs. In Figure 6b, the double reciprocal plot was linear and used to calculate the *K_m_* value from the slope and *V_max_* from the y-intercept using Equation (3).

(3) $$\frac{1\;}{V_{0}}=\frac{\;K_{m}}{V_{max}}.\frac{1}{\left[S\right]}+\frac{\;1}{V_{max}}$$

where *V*_0_ is the initial rate, *V_max_* is the maximum reaction rate, [*S*] is the concentration of the substrate (H_2_O_2_), and *K_m_* is the Michaelis–Menten constant. The affinity between the enzyme and the substrate is defined by *K*_m_. The higher the *K*_m_, the weaker the interaction between the enzyme and substrate. A low *K_m_* value indicates a strong interaction between the substrate and catalyst. The apparent Michaelis–Menten constant (*K_m_*) for H_2_O_2_ was found to be 7.09 mM. This is the concentration at which the reaction rate reaches half of its maximum value. The maximum velocity (*V_max_*) was calculated to be 4.70 × 10^−8^ M^−1^.s. The average *K_m_* value suggests good interaction of PVP-NiONPs toward H_2_O_2_. This is good for effective ROS generation and OPD oxidation can occur when H_2_O_2_ is present in relatively low concentrations. For the OPD calculation from graphs in Figure 6c,d, the *K_m_* and *V_max_* values obtained were 0.59 mM and 4.03 × 10^−8^ M^−1^.s, respectively. The much lower *K_m_* value obtained for OPD indicates excellent interaction between PVP-NiONPs and OPD for optimal OPD oxidation by ROS. These findings were also observed for other reported metal oxide nanomaterials which rely on surface-mediated electron transfer or catalytic processes rather than substrate-specific binding in natural enzymes [47]. For comparison with other nanozymes in Table 1, the PVP-NiONPs have very low *K_m_* values and the highest *V_max_* values. This shows that PVP-NiONPs performed much better.

### 3.6. Confirming Reaction Oxygen Radical Species (ROS) Generation

**Figure 7:**
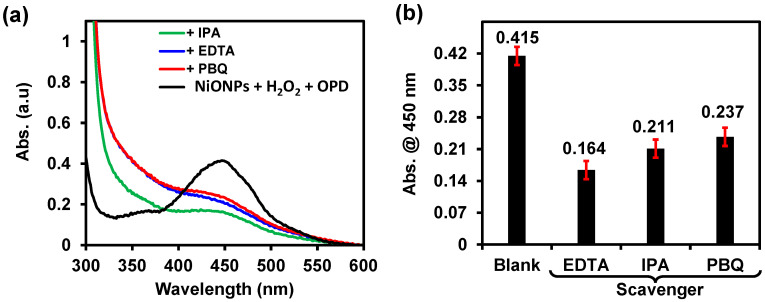
(**a**) UV-vis spectra of the oxidation of OPD to coloured products with (NiONPs + H_2_O_2_ + OPD) with addition of radical scavengers (+ EDTA, + IPA, and + PBQ) and (**b**) the corresponding bar graph.

The absorption of oxidized OPD was investigated in a solution containing PVP-NiONPs and H_2_O_2_. The catalytic reaction of PVP-NiONPs and H_2_O_2_ generates reactive oxygen radical species (h^+^, HO^●─^, HO_2_**^●^**^─^ and O_2_^●─^). ROS generation was investigated using a scavenger experiment to capture the radical species and evaluate the impact of each radical. The radical scavengers investigated are EDTA, IPA and PBQ, known to scavenge h^+^, HO^●─^ and O_2_^●─^, respectively. Figure 7a shows the effect before and after adding a scavenger in the reaction containing PVP-NiONPs, H_2_O_2_, and OPD. The absorption of the oxidized OPD was monitored at 450 nm. Figure 7b shows the absorbance of oxidized OPD in the absence of a scavenger with the average absorbance value of 0.415 a.u and the presence of a scavenger which resulted in the decrease in the absorption of OPD due to the scavenger. The average absorbance values in the presence of the scavenger were 0.164 a.u (EDTA), 0.211 a.u (IPA), and 0.237 a.u (PBQ).

The absorption values at 450 nm decreased when the scavenger was added. The highest decrease (60.5%) was observed for EDTA, specifically for h^+^. This was followed by the 49.2% decrease when IPA was added to scavenge hydroxyl radicals, HO^●─^, and lastly the PBQ scavenged peroxyradicals (O_2_^●─^) with about 42.9%. Thus, the mechanism of peroxidase-like activity of PVP-NiONPs proceeded via catalytic reduction of H_2_O_2_ to generate reactive oxygen radical species that catalyze the oxidation of OPD to yellow-coloured products (2,3-diaminophenazine).

### 3.7. Colorimetric Detection of Ascorbic Acid

**Figure 8:**
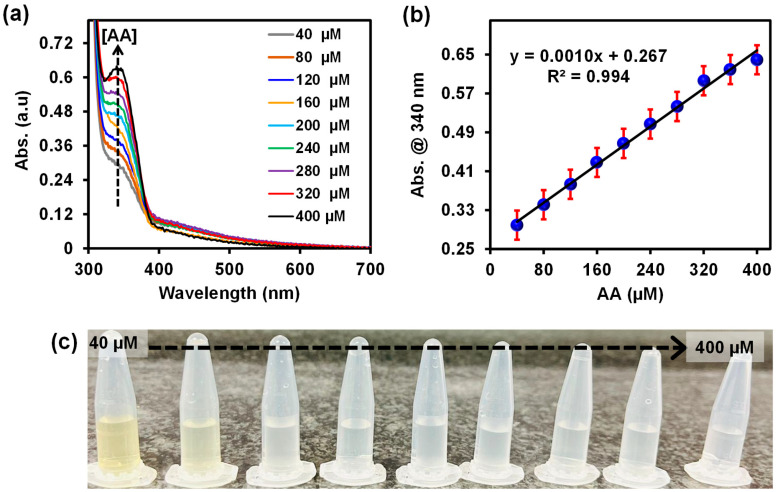
(**a**) UV-vis spectra and (**b**) corresponding linear calibration curve. (**c**) Images showing colour changes at the increase in concentration of ascorbic acid from 40 µM to 400 µM.

Ascorbic acid detection was followed by the reduction of the oxidized OPD (DAP) to form 2,3-diamino-5,10-dihydrophenazine (DADHP) using UV-vis spectroscopy and absorption at 340 nm. The detection of AA in Figure 8a show UV-vis spectra. ROS generated by PVP-NiONPs and H_2_O_2_ oxidize OPD to form 2,3-diaminophenazine (DAP). In the presence of AA, the oxidized OPD products are reduced to 2,3-diamino-5,10-dihydrophenazine (DADHP). The increase in the absorption peak at 340 nm is due to the formation of DADHP and can be linearly related to the concentration of AA as shown in Figure 8b. Figure 8c shows the images of solutions upon adding varying concentrations of ascorbic acid. The intensity of absorption at 340 nm was found to be proportional to the ascorbic acid concentration from 40 µM to 400 µM. The linear regression Equation (4) of the absorbance graph versus varied ascorbic acid concentration was:

Abs. @340 nm = 0.0010 [AA] + 0.267(4)

with an R^2^ value of 0.994. The linear relation suggests that this colorimetric system provides a reliable response in the concentration range investigated. The correlation coefficient corresponds to excellent linearity and minimal deviation. Moreover, it validates the applicability of this sensor for the analytical detection of AA. The limit of detection (LOD) and limit of quantification (LOQ) were calculated using the slope of the regression line and standard deviation of the blank, and the values obtained were 0.11 µM using 3σ and 0.36 µM using 10σ respectively. This reaction system is highly sensitive and reproducible. Similar observations were found in the study of Isho et al. [25].

The peak due to DAP at 450 nm was not observed and this was due to the instant reduction of DAP to form DADHP with an absorption at 340 nm. Similar observations were reported by Yan et al. [52]. They observed that ascorbic acid has antioxidant and redox properties that enable it to immediately reduce DAP to DADHP as shown in Scheme 1. This was observed for the oxidation of 3,3′,5,5′-tetramethylbenzidine (TMB) to a blue-coloured product, thus preventing colour change. For the blank reaction, the absorption of oxidized OPD occurs in the presence of PVP-NiONPs and H_2_O_2_ to form 2,3-diaminophenazine (DAP) with a measurable peak at 450 nm [53]. When a different concentration of AA is introduced, it results in the reduction of oxidized OPD to form DADHP with a wavelength at 340 nm. Compared with other nanozymes for sensing ascorbic acid, the proposed colorimetric method using PVP-NiONPs exhibited a better limit of detection and limit of quantification in a linear range as summarized in Table 2.

### 3.8. Selectivity, Reproducibility, and Applicability

**Figure 9:**
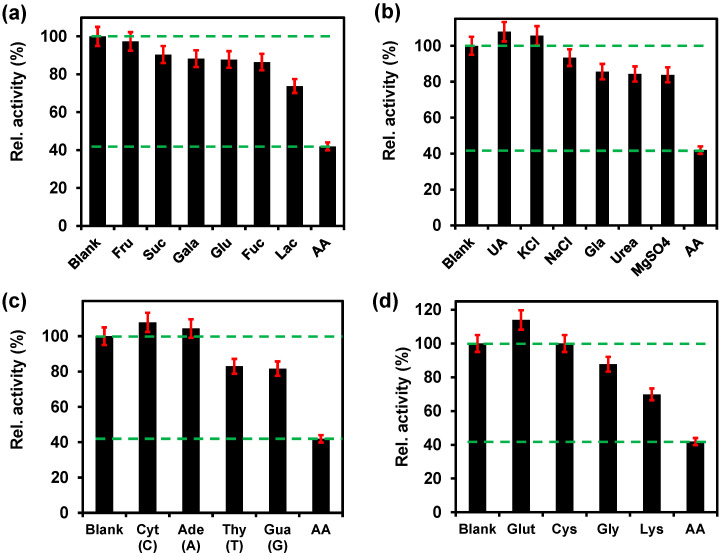
Selectivity of the proposed colorimetric sensor based on NiONPs towards (**a**) carbohydrates, (**b**) organic compounds and inorganic salts, (**c**) nucleobases, and (**d**) amino acids.

The selectivity of the developed ascorbic acid sensor was evaluated by investigating its response to other closely related biomolecules. The interfering species included metal ions, reducing agents, carbohydrates and amino acids. Each of the interfering species was individually added instead of ascorbic acid under identical experimental conditions to assess any non-specific interactions. The control or blank solution, containing PVP-NiONPs, H_2_O_2_ and OPD, resulted in a colour change to yellow and high absorption intensity at 450 nm due to the oxidation of OPD as shown in Figure 9. The relative activity of the blank was treated as 100%. After adding AA, there was a dramatic decrease in the absorption intensity at 450 nm to about 41.0% and there was no colour. The majority of the interfering species resulted in an increase in intensity at 450 nm and colour changes to yellow similar to the control or blank solution, signifying no interference. When the relative reactivity was investigated as shown in Figure 9, glutathione (Glut, 113.9%), adenine (Ade, 104.4%), cytosine (Cyt, 107.9%), uric acid (UA, 107.9%) and KCl (105.7%) exhibited an absorption intensity slightly higher than the control or blank solution. This signifies that these compounds induce some oxidation properties towards OPD. Lysine showed a decrease in absorption intensity to 69.9%, still much higher than the 41.9% for AA. This can be attributed to the fact that it has two amine groups that undergo weak electron transfer and exhibit electrochemical activity [59] that allows it to partially convert oxidized OPD to its reduced form. Furthermore, it is known to be a weak antioxidant in nature and act as a radical scavenger to reduce hydroxyl radicals [60]. This suggests that the proposed sensor is highly selective as it selectively detects AA even in the presence of other reducing agents.

The colorimetric sensing of ascorbic acid was further investigated in 10% vitamin C tablets purchased at a Grahamstown Pharmacy, representing real sample. The 10% vitamin C solution was prepared in pH 4.0 acetate buffer spiked with known concentrations of ascorbic acid within the calibration curve in Figure 8. Table 3 shows the % recovery of ascorbic acid in 10% vitamin C tablets was between 92.3% and 102.6% falling within acceptable analytical limits. The percentage relative standard deviation (%RSD) values were between 0.46% and 1.25%, indicating the high precision and reproducibility of this method. The results showed the feasibility and reliability of the proposed PVP-NiONPs, OPD and H_2_O_2_ combination as the colorimetric sensor for detecting ascorbic acid in 10% vitamin C.

## 4. Conclusions

The preparation of polyvinylpyrrolidone nickel oxide nanoparticles was successful and characterized using various spectroscopic and microscopic techniques to confirm their preparation. The PVP-NiONP particle sizes were found to be 9.6 nm using XRD, 13.5 ± 3.7 nm using DLS and 9.5 ± 3.5 nm using TEM. The nanomaterials also showed the presence of oxygen vacancy (useful for catalysis) as showed by Raman spectroscopy. The PVP-NiONPs exhibited excellent peroxidase-like activity using OPD and H_2_O_2_ as substrates. A typical Michaelis−Menten enzyme behaviour was observed for the PVP-NiONPs using steady-state kinetic analysis which showed good affinity towards H_2_O_2_ and OPD compared to other reported nanozymes. The PVP-NiONPs were utilized for the detection of ascorbic acid with a wide linear concentration range between 40 μM and 400 μM. The limit of detection and limit of quantification were determined to be 0.11 μM and 0.36 μM respectively. Selectivity towards ascorbic acid compared to potential interfering species was achieved. PVP-NiONPs applied in a real sample analysis using 10% vitamin C tablets confirmed the reliability for ascorbic acid detection in real-life applications. The % recovery of ascorbic acid in 10% vitamin C was between 92.3% and 102.6% and the %RSD values were between 0.46% and 1.25% indicating the high precision and reproducibility of this method.

## Figures and Tables

**Table 1:** Summary of the kinetic parameters (*K*_m_ and *V*_max_) for the substrates (H_2_O_2_ and OPD) with PVP-NiONPs, HRP and other reported nanozymes. TW: This Work.

Nanozymes	*K_m_* (mM)		*V_max_* (×10^−8^ M.s^−1^)	
	H_2_O_2_	OPD	H_2_O_2_	OPD
PVP-NiONPs^TW^	7.09	0.59	4.70	4.03
BSA-MnO_2_ NPs [48]	0.12	0.31	5.71	8.21
HRP [48]	0.34	0.59	9.48	4.65
cc-AuNPs [49]	159.7	0.69	59.7	45.4
ZnCo_2_O_4_ nanosheet [50]	0.23	0.40	4.75	5.90
CuO/WO_3_-GO [51]	277	0.152	19.8	15.8
CoO-GO [52]	773	0.328	19.7	13.0

**Table 2:** Comparison of PVP-NiONPs for colorimetric detection of ascorbic acid with reported nanozyme-based sensors. TW: This Work. ^a^ colorimetric and ^b^ fluorescence.

Nanozymes	Linear Range (µM)	LoD (µM)	LoQ (µM)
PVP-NiONPs^TW^	40–400	0.11	0.36
CoNi_2_O_4_ [21]	1–140	0.44	-
AuNCs [54]	0.5–200	0.22 ^a^	-
		0.15 ^b^	-
AgNPs [55]	0–100	0.69 ^a^	
		0.17 ^b^	
M.CQDs [56]	10–70	3.36	-
CuMnO_2_ nanoflowers [57]	1–105	0.39	-
HAp@Fe_2_O_3_ [58]	0.6–56	0.16	0.53

**Table 3:** Summary of the analytical results of the amount of AA recovered in the commercial 10% vitamin C tablets dissolved in pH 4.0 acetate buffer solution.

Added [AA] (µM)	Detected [AA] (µM)	Recovery (%)	RSD (%)
120	111.6	98.0	0.66
160	147.6	92.3	1.25
200	205.1	102.6	0.46

## Data Availability

The original contributions presented in this study are included in the article/supplementary material. Further inquiries can be directed to the corresponding author.

## Supplementary Materials

The following supporting information can be downloaded at: https://www.mdpi.com/article/10.3390/bios16050299/s1, Figure S1: UV-vis spectra showing the effect of reaction conditions on the peroxidase-like activity of PVP-NiONPs in a solution containing OPD and H_2_O_2_. (a) pH (2.0–8.0), (b) reaction time (2 min–20 min), and (c) temperature (20 °C–70 °C).
