# Supplementary material for: Catalytic Reduction of H2O2 by Polyvinylpyrrolidone Nickel Oxide Nanozymatic Activity and Colorimetric Sensing of Ascorbic Acid
Source: Biosensors (Basel). 2026 May 21;16(5):299. doi: 10.3390/bios16050299 (PMC13204353; doi:10.3390/bios16050299)
Supplement: Supplementary file 1 [file biosensors-16-00299-s001.zip › biosensors-4266648-supplementary.pdf]

biosensors

Supplementary Information (SI)

Catalytic Reduction of H_2O_2 by Polyvinylpyrrolidone Nickel Oxide Nanozymatic Activity and Colorimetric Sensing of Ascorbic Acid

Mosebudi Rambevha, Ridge Chavalala and Philani Mashazi *

Institute for Nanotechnology Innovation, Rhodes University, P.O. Box 94, Makhanda 6140, South Africa; g24r5071@campus.ru.ac.za (M.R.); g17c9530@campus.ru.ac.za (R.C.)

* Correspondence: p.mashazi@ru.ac.za; Tel.: +27-46-603-8846

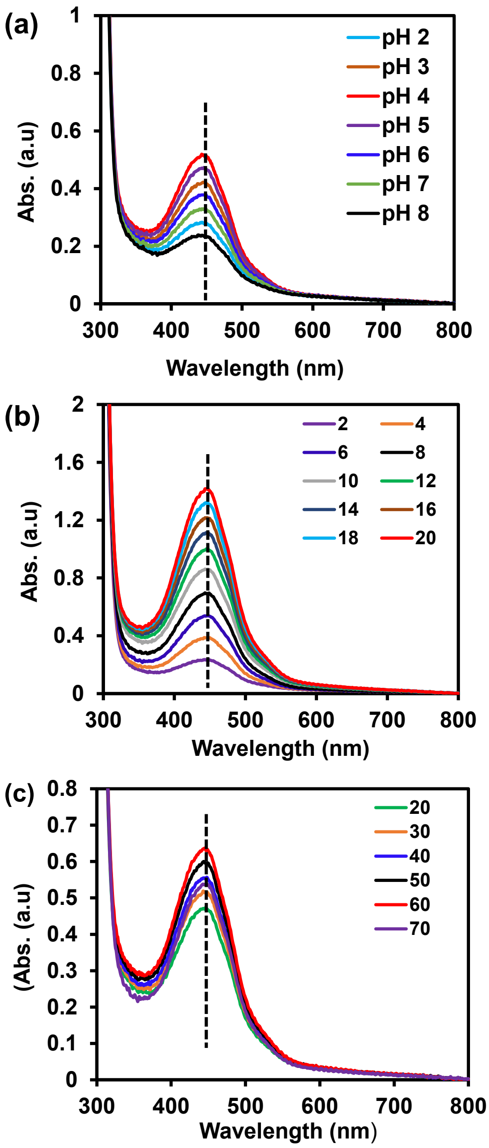

Figure S1. UV-vis spectra showing the effect of reaction conditions on the peroxidase-like activity of PVP-NiONPs in a solution containing OPD and H_2O_2. (**a**) pH (2.0 – 8.0), (**b**) reaction time (2 min – 20 min), and (**c**) temperature (20⁰C – 70⁰C).
